# Clinically Relevant Discordance Between SCORE2 and REGICOR in Spanish Workers: Implications for Cardiovascular Risk Reclassification in Primary Prevention

**DOI:** 10.3390/medsci14020294

**Published:** 2026-06-06

**Authors:** Manuel Sarmiento Cruz, Pedro Juan Tárraga López, Mónica Silu Piña Dabreu, Lluis Rodas Cañellas, Ángel Arturo López-González, José Ignacio Ramírez-Manent

**Affiliations:** 1 Primary Care, Balearic Islands Health Service, 07010 Palma, Spain; msarmiento1979@gmail.com (M.S.C.); joseignacio.ramirez@ibsalut.es (J.I.R.-M.); 2 Faculty of Medicine, University of Castilla La Mancha (UCLM), 02008 Albacete, Spain; pedrojuan.tarraga@uclm.es; 3 ADEMA-University School, University of the Balearic Islands, 07009 Palma, Spain; m.pina@eua.edu.es (M.S.P.D.); ll.rodas@eua.edu.es (L.R.C.)

**Keywords:** SCORE2, REGICOR, cardiovascular risk, primary prevention, occupational health, risk reclassification

## Abstract

Background: Cardiovascular risk prediction models are central to primary prevention strategies, yet substantial variability exists between contemporary and traditional equations used in clinical practice. In Spain, SCORE2 and REGICOR currently coexist as major cardiovascular risk assessment tools despite important methodological differences. However, evidence regarding their concordance in large occupational populations remains limited. Objective: To evaluate the agreement between SCORE2 and REGICOR in cardiovascular risk stratification among Spanish workers and to quantify the extent of cardiovascular risk reclassification associated with SCORE2 implementation. Methods: A multicenter cross-sectional study was conducted in 216,310 Spanish workers aged 40–64 years undergoing routine occupational health examinations between 2019 and 2024. Cardiovascular risk was estimated using SCORE2, REGICOR, ERICE, DORICA, Globorisk, and Framingham-based equations. High-risk categories were defined according to the thresholds recommended for each model. Agreement between categorical classifications was assessed using Cohen’s kappa coefficient, whereas Pearson correlation coefficients were calculated for continuous risk estimates. Results: SCORE2 classified 15,617 workers (7.22%) as high cardiovascular risk, whereas REGICOR identified only 4409 individuals (2.04%). Among workers classified as high risk by SCORE2, 14,387 (92.1%) were not identified as high risk by REGICOR. Agreement between SCORE2 and REGICOR was slight (kappa = 0.094), indicating minimal concordance in high-risk classification. By contrast, SCORE2 demonstrated higher agreement with Framingham hard coronary events (kappa = 0.567) and Globorisk (kappa = 0.534). Correlation analyses showed strong associations between SCORE2 and several continuous cardiovascular risk estimates, including the Framingham categorical score (r = 0.768), Framingham hard coronary events (r = 0.758), and Globorisk (r = 0.739), whereas the correlation between SCORE2 and REGICOR was substantially lower (r = 0.251). These findings indicate that strong statistical correlation does not necessarily translate into clinically meaningful agreement in cardiovascular risk categorization. Conclusions: Substantial discordance exists between SCORE2 and REGICOR in the identification of high cardiovascular risk among Spanish workers. SCORE2 consistently classified a considerably larger proportion of individuals as high risk, whereas REGICOR showed limited concordance with contemporary cardiovascular prediction models. Continued reliance on REGICOR instead of SCORE2 may lead to under-identification of workers who could currently be considered candidates for intensified primary cardiovascular prevention according to contemporary European prevention strategies. Nevertheless, the present study does not establish which model provides superior prediction of future cardiovascular events, and prospective outcome-based validation studies remain necessary.

## 1. Introduction

Cardiovascular disease (CVD) remains the leading cause of mortality worldwide and continues to represent one of the greatest challenges for contemporary health systems despite substantial advances in prevention and treatment strategies. According to the Global Burden of Disease Study 2019, cardiovascular diseases account for nearly one-third of all global deaths, with ischemic heart disease and stroke representing the predominant causes of disability-adjusted life years and premature mortality worldwide. In Europe, and particularly in aging populations, the burden associated with cardiovascular morbidity extends beyond mortality itself, contributing substantially to healthcare utilization, productivity loss, and socioeconomic impact. Consequently, the accurate identification of individuals at elevated cardiovascular risk remains a cornerstone of primary prevention strategies and clinical decision-making [1,2].

Current preventive approaches are largely based on the estimation of global cardiovascular risk through multivariable prediction models integrating traditional risk factors such as age, sex, smoking status, blood pressure, and lipid profile. These tools play a central role in guiding therapeutic interventions, including lipid-lowering therapy, antihypertensive treatment, and the intensity of lifestyle modification strategies. In recent decades, cardiovascular risk estimation has progressively evolved from a predominantly epidemiological concept into a practical clinical instrument that directly influences individualized preventive care [3,4].

However, the coexistence of multiple cardiovascular risk prediction models poses important challenges in routine clinical practice. Different equations have been developed using heterogeneous populations, distinct cardiovascular outcomes, and diverse calibration strategies, resulting in potentially substantial discrepancies in estimated risk for the same individual. As many therapeutic recommendations are linked to predefined cardiovascular risk thresholds, variability between models may lead to clinically relevant differences in treatment eligibility and preventive intensity [5].

In Spain, cardiovascular risk assessment has historically relied on adaptations of the Framingham equations. Among these, the REGICOR function has become one of the most widely implemented tools in both primary care and occupational health settings. REGICOR was developed through recalibration of the original Framingham coronary risk equation to the Spanish population in order to improve risk estimation in Mediterranean populations characterized by lower coronary event rates than those observed in North American cohorts. Previous investigations demonstrated improved calibration compared with the original Framingham model in low-risk populations, supporting its widespread incorporation into clinical practice [6,7,8]. Nevertheless, concerns have persisted regarding the potential underestimation of cardiovascular risk in certain patient subgroups, particularly among individuals with multiple cardiovascular risk factors, diabetes, or subclinical atherosclerosis [9].

The European Society of Cardiology (ESC) introduced a major change in cardiovascular prevention strategies with the publication of the 2021 guidelines and the implementation of SCORE2 as the new recommended model for cardiovascular risk estimation in European populations aged 40–69 years without established cardiovascular disease. Unlike the original SCORE algorithm, SCORE2 estimates the 10-year risk of both fatal and non-fatal cardiovascular events and incorporates recalibration according to regional cardiovascular risk profiles across Europe. The model was developed and validated using contemporary European cohorts, including more than 600,000 individuals and was intended to improve risk prediction in current clinical settings characterized by declining cardiovascular mortality and changing epidemiological patterns [10,11].

The transition from previous cardiovascular risk functions to SCORE2 may have important clinical implications because different models can classify the same patient into substantially different risk categories. Emerging evidence suggests that SCORE2 may identify a larger proportion of individuals as candidates for intensive preventive interventions compared with previously used scales. Furthermore, recent external validation studies have highlighted important variability in SCORE2 performance across different populations and socioeconomic contexts, reinforcing the concept that cardiovascular risk estimation remains highly dependent on population characteristics and local calibration [12,13,14].

Despite the incorporation of SCORE2 into the ESC prevention guidelines and its subsequent adoption in national recommendations such as the 2024 update of the Programa de Actividades Preventivas y de Promoción de la Salud (PAPPS), REGICOR continues to be widely used in Spanish clinical practice and occupational medicine settings [15]. This coexistence of different cardiovascular risk assessment tools raises a clinically relevant question: to what extent do SCORE2 and REGICOR classify the same individuals as being at high cardiovascular risk?

This issue may be particularly relevant in occupational populations. Workers represent a specific epidemiological subgroup influenced by the so-called healthy worker effect, a phenomenon describing the tendency of employed individuals to exhibit lower overall morbidity and mortality than the general population [16]. Nevertheless, cardiovascular disease remains a major contributor to work disability, reduced productivity, and long-term occupational burden, emphasizing the importance of accurate cardiovascular prevention strategies in this setting [17].

Previous studies comparing cardiovascular risk functions in Spain mainly focused on SCORE, Framingham-derived equations, and REGICOR, reporting variable and often limited agreement between scales [18,19,20]. However, evidence regarding the concordance between SCORE2 and REGICOR in large contemporary Spanish working populations remains scarce. Moreover, most previous investigations primarily evaluated statistical correlation between scales rather than clinically meaningful reclassification of high-risk individuals.

Importantly, correlation between continuous risk estimates does not necessarily imply agreement in clinical decision-making. Two cardiovascular risk models may exhibit a relatively strong linear association while simultaneously classifying markedly different individuals into high-risk categories, potentially leading to divergent therapeutic recommendations. From a clinical perspective, discrepancies in high-risk classification may therefore be more relevant than correlation coefficients alone.

Accordingly, the aim of the present study was to evaluate the agreement between SCORE2 and REGICOR in cardiovascular risk stratification in a large cohort of Spanish workers. Additionally, we sought to quantify the extent of cardiovascular risk reclassification associated with SCORE2 implementation and to compare SCORE2 with other commonly used cardiovascular risk models.

## 2. Methods

### 2.1. Study Design and Population

A cross-sectional multicenter study was conducted in a large cohort of Spanish workers undergoing routine occupational health examinations between January 2019 and December 2024. Participants were recruited through occupational risk prevention services located in several Spanish autonomous communities and representing different occupational sectors, including healthcare, industry, construction, transport, administration, and service-related activities.

The initial study population included 217,793 actively employed workers aged between 40 and 64 years. Individuals with missing clinical or biochemical information required for cardiovascular risk estimation were excluded from the final analysis. After data quality assessment and exclusion of incomplete records, 216,310 participants were ultimately included.

Occupational medical examinations were performed according to standardized workplace health surveillance protocols established under Spanish occupational health legislation. These evaluations included structured clinical interviews, physical examination, anthropometric assessment, blood pressure measurements, and laboratory testing.

The study was conducted in accordance with the principles established in the Declaration of Helsinki and complied with current European and Spanish regulations regarding biomedical research and personal data protection [21,22,23]. The database used for the present analysis belonged to a previously approved epidemiological research project evaluated by an institutional ethics committee.

### 2.2. Clinical and Laboratory Variables

Demographic, anthropometric, clinical, and biochemical variables required for cardiovascular risk estimation were collected during occupational health assessments. These variables included age, sex, smoking status, systolic and diastolic blood pressure, body mass index, total cholesterol, high-density lipoprotein cholesterol (HDL-C), low-density lipoprotein cholesterol (LDL-C), triglycerides, and fasting plasma glucose.

Anthropometric measurements were obtained by trained healthcare professionals following standardized procedures. Body weight was measured to the nearest 0.1 kg using a calibrated digital scale SECA 700 (SECA, Chino, CA, USA), with participants wearing light clothing and no shoes. Height was measured to the nearest 0.1 cm using a wall-mounted stadiometer SECA 220 (SECA, Chino, CA, USA). Body mass index (BMI) was calculated as weight (kg) divided by height squared (m^2^) and classified according to World Health Organization criteria [24].

Blood pressure measurements were obtained under resting conditions using validated sphygmomanometers OMRON-M3 monitor (OMRON, Osaka, Japan) following current European recommendations for hypertension assessment [25]. When more than one blood pressure measurement was available, the mean value was used for analysis.

Venous blood samples were collected after overnight fasting and analyzed in certified clinical laboratories using standardized analytical methods routinely applied in occupational medicine practice [26]. Smoking status was categorized dichotomously as current smoker or non-smoker according to self-reported tobacco consumption obtained during the clinical interview.

### 2.3. Cardiovascular Risk Estimation

Cardiovascular risk was estimated using several validated cardiovascular risk prediction models commonly applied in European and Spanish clinical practice, including SCORE2, REGICOR, ERICE, DORICA, Globorisk, and Framingham-based equations. The primary analysis focused on the comparison between SCORE2 and REGICOR because these models currently represent two conceptually different approaches to cardiovascular risk assessment that coexist in routine clinical practice in Spain.

SCORE2 estimates the 10-year probability of fatal and non-fatal cardiovascular disease in individuals without previous cardiovascular disease or diabetes and incorporates recalibration according to regional cardiovascular risk profiles across Europe. In the present study, SCORE2 was calculated using the low-risk European calibration recommended for Spain by the 2021 European Society of Cardiology prevention guidelines [10,27].

In accordance with the original SCORE2 recommendations, cardiovascular risk estimation was restricted to individuals without established cardiovascular disease. Information regarding previous cardiovascular disease was obtained from the occupational medical history and clinical interview conducted during routine health examinations. Participants with a documented history of cardiovascular disease were not eligible for SCORE2 assessment. Diabetes status was recorded as part of the routine occupational health evaluation. The implementation of each cardiovascular risk equation followed the recommendations and eligibility criteria established in the corresponding original publications whenever the required variables were available.

REGICOR corresponds to a calibrated adaptation of the original Framingham coronary risk equation developed specifically for the Spanish population. The model was designed to improve cardiovascular risk estimation in Mediterranean populations characterized by lower cardiovascular incidence rates than those observed in North American cohorts [6,28].

Additional cardiovascular risk scales were analyzed as secondary comparators in order to evaluate the consistency of cardiovascular risk classification across different predictive strategies currently used in preventive cardiology and epidemiological research.

High cardiovascular risk categories were defined according to the thresholds recommended for each specific model. For REGICOR and Framingham-derived equations, high cardiovascular risk was defined as a predicted 10-year risk ≥10% [29]. For SCORE2, high-risk classification followed the age-specific thresholds proposed by the 2021 ESC prevention guidelines for low-risk European countries (≥2.5% in individuals younger than 50 years and ≥5% in those aged 50–69 years) [4,10].

These thresholds were selected because they represent the cut-off values currently used to guide the intensification of preventive cardiovascular interventions in contemporary European clinical practice.

### 2.4. Statistical Analysis

Continuous variables are presented as mean and standard deviation, whereas categorical variables are expressed as absolute frequencies and percentages.

The primary outcome of the study was the agreement between SCORE2 and REGICOR in the classification of individuals with high cardiovascular risk. Agreement between categorical classifications was evaluated using Cohen’s kappa coefficient. Kappa values were interpreted according to the classification proposed by Landis and Koch as follows: values below 0.20 were considered slight agreement, values between 0.21 and 0.40 fair agreement, values between 0.41 and 0.60 moderate agreement, values between 0.61 and 0.80 substantial agreement, and values above 0.80 almost perfect agreement [30].

To further characterize the relationship between scales, Pearson correlation coefficients were calculated using the continuous risk estimates generated by each cardiovascular risk model. Nevertheless, because several risk equations share common predictive variables, correlation analyses were interpreted cautiously, recognizing that a strong linear association does not necessarily imply clinically meaningful concordance [31].

Cross-classification analyses were additionally performed to quantify the proportion of individuals classified as high cardiovascular risk by SCORE2 but not by REGICOR, representing the subgroup potentially reclassified under contemporary European prevention recommendations.

Missing data represented less than 1% of the total dataset for all variables included in cardiovascular risk estimation. Therefore, complete-case analysis was performed without multiple imputation procedures [32].

All statistical analyses were conducted using IBM SPSS Statistics version 30.0 (IBM Corp., Armonk, NY, USA). A two-sided *p* value < 0.05 was considered statistically significant.

## 3. Results

### 3.1. Study Population and Baseline Characteristics

After applying the predefined eligibility criteria and excluding individuals with incomplete information required for cardiovascular risk estimation, a total of 216,310 workers aged 40 to 64 years were included in the final analysis. Men represented 60.7% of the study population (*n* = 131,260), whereas women accounted for 39.3% (n = 85,050). The mean age of the overall cohort was 48.8 ± 6.2 years.

**Figure 1:**
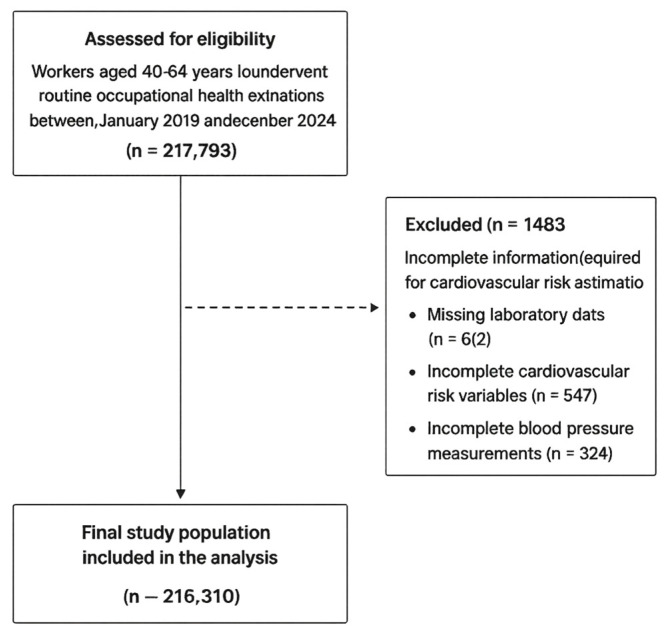
Flowchart of participant selection and final study population.

The selection process of the study population and the reasons for exclusion are summarized in Figure 1.

Flowchart showing the selection process of the study population included in the analysis. A total of 217,793 workers aged 40–64 years undergoing routine occupational health examinations were initially assessed. After exclusion of individuals with incomplete clinical or biochemical information required for cardiovascular risk estimation, 216,310 participants were included in the final analysis.

The overall prevalence of current smoking was 32.5%, with very similar proportions in men and women. The mean systolic and diastolic blood pressure values were 127.6 ± 17.2 mmHg and 78.8 ± 11.1 mmHg, respectively. The mean body mass index was 27.0 ± 4.7 kg/m^2^, consistent with an overall overweight population profile.

Lipid parameters also reflected an unfavorable cardiometabolic profile in a substantial proportion of the cohort. Mean total cholesterol concentration was 204.0 ± 36.7 mg/dL, mean HDL cholesterol was 51.3 ± 9.3 mg/dL, and mean LDL cholesterol was 129.0 ± 34.9 mg/dL. Median triglyceride concentration was 101 mg/dL, with an interquartile range of 74 to 143 mg/dL. Mean fasting glucose was 94.8 ± 22.1 mg/dL.

Sex-related differences were observed in several cardiovascular risk factors. Men showed higher systolic and diastolic blood pressure values, body mass index, triglycerides, and fasting glucose concentrations, whereas women exhibited higher HDL cholesterol levels. Total cholesterol and LDL cholesterol values were comparable between sexes. Baseline demographic, clinical, anthropometric, and biochemical characteristics of the study population according to sex are summarized in Table 1.

### 3.2. Distribution of Continuous Cardiovascular Risk Estimates

Continuous cardiovascular risk estimates varied substantially across the different prediction models. The mean SCORE2 estimated risk was 1.25 ± 1.96%, with a median value of 1.0%. By contrast, the mean REGICOR estimated risk was 3.29 ± 2.30%, with a median value of 3.0%. Although REGICOR yielded numerically higher continuous percentage estimates than SCORE2, this did not translate into a higher proportion of individuals classified as high cardiovascular risk, reflecting the different endpoints, calibration methods, and clinical thresholds used by each model.

Among the remaining risk functions, mean estimated risk values were 5.22 ± 5.12% for ERICE, 6.28 ± 4.92% for DORICA, 7.62 ± 6.15% for the Framingham categorical score, 5.29 ± 5.21% for the Framingham hard coronary events score, and 4.21 ± 3.26% for Globorisk.

Several models showed asymmetric distributions, with median values lower than mean estimates.

This heterogeneity in continuous risk estimates illustrates that the absolute numerical values generated by different models are not directly interchangeable, even when calculated in the same individuals.

Continuous cardiovascular risk estimates varied substantially across the different prediction models evaluated in the study population, as shown in Table 2.

Although several models yielded relatively similar median risk estimates, substantial variability was observed in the distribution and upper ranges of predicted cardiovascular risk across scales, particularly among Framingham-derived equations and DORICA.

### 3.3. Prevalence of High Cardiovascular Risk According to Each Model

The proportion of individuals classified as having high cardiovascular risk differed markedly according to the model used. SCORE2 classified 15,617 workers as high risk, corresponding to 7.22% of the study population. REGICOR identified 4409 workers as high risk, corresponding to only 2.04% of the cohort.

Thus, SCORE2 classified approximately 3.5 times more individuals as high cardiovascular risk than REGICOR.

Globorisk showed a prevalence of high cardiovascular risk similar to SCORE2, identifying 16,050 individuals as high risk (7.42%). ERICE classified 12,632 individuals as high risk (5.84%), whereas DORICA classified 5405 individuals as high risk (2.50%).

The highest proportion of high-risk individuals was observed with the Framingham categorical score, which classified 63,309 participants as high risk (29.27%). The Framingham hard coronary events score identified 25,001 individuals as high risk (11.56%).

**Figure 2:**
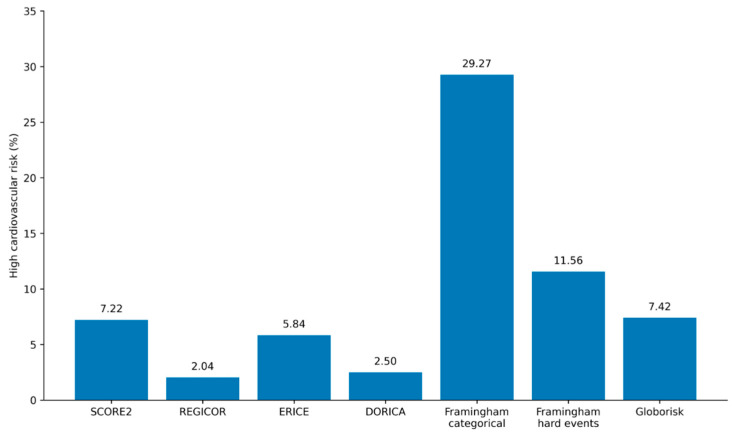
Prevalence of high cardiovascular risk according to different cardiovascular risk prediction models.

Marked differences were observed in the proportion of individuals classified as high cardiovascular risk according to the prediction model used (Figure 2).

The figure shows the percentage of workers classified as having high cardiovascular risk according to each cardiovascular risk prediction model. Substantial variability was observed across scales, ranging from 2.04% with REGICOR to 29.27% with the Framingham categorical score. SCORE2 identified approximately 3.5 times more high-risk individuals than REGICOR.

The proportion of workers classified as having high cardiovascular risk varied markedly depending on the cardiovascular risk model applied, as detailed in Table 3.

Substantial heterogeneity was observed across cardiovascular risk prediction models. SCORE2 identified more than three times as many high-risk individuals as REGICOR, whereas the Framingham categorical score yielded the highest proportion of high-risk classifications in the overall cohort.

### 3.4. Discordance Between SCORE2 and REGICOR in High-Risk Classification

The cross-classification between SCORE2 and REGICOR revealed a marked discordance in the identification of high-risk individuals. Overall, 197,514 participants were classified as non-high risk by both models, whereas only 1230 participants were classified as high risk by both SCORE2 and REGICOR.

Among the 15,617 individuals classified as high risk by SCORE2, 14,387 were not classified as high risk by REGICOR. This means that 92.1% of workers considered high risk by SCORE2 would not have been identified as high risk using REGICOR.

Conversely, among the 4409 individuals classified as high risk by REGICOR, 3179 were not classified as high risk by SCORE2, corresponding to 72.1% of REGICOR high-risk individuals.

From the overall cohort perspective, 6.65% of all workers were classified as high risk by SCORE2 but not by REGICOR, whereas 1.47% were classified as high risk by REGICOR but not by SCORE2. The proportion classified as high risk by both models was only 0.57%.

Although the crude overall percentage agreement between SCORE2 and REGICOR was high (91.9%), this finding was largely driven by the predominance of individuals classified as non-high risk by both models. The low number of participants jointly classified as high risk highlights the limited clinical overlap between both tools for identifying individuals who may be candidates for intensified cardiovascular prevention.

Cross-classification analysis between SCORE2 and REGICOR demonstrated substantial discordance in the identification of high cardiovascular risk individuals, as shown in Table 4.

Among the 15,617 workers classified as high cardiovascular risk by SCORE2, 14,387 (92.1%) were not identified as high risk by REGICOR. Conversely, only 1230 individuals were jointly classified as high risk by both models, highlighting the limited clinical overlap between SCORE2 and REGICOR.

**Figure 3:**
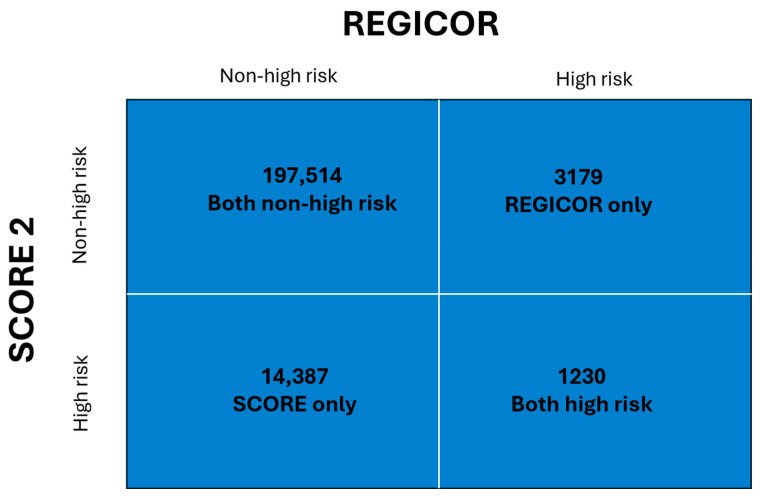
Reclassification pattern between SCORE2 and REGICOR for high cardiovascular risk identification. Cross-classification of workers according to high cardiovascular risk categories defined by SCORE2 and REGICOR. The figure illustrates the limited overlap between both models in identifying high-risk individuals. A total of 14,387 workers were classified as high cardiovascular risk by SCORE2 but not by REGICOR, whereas only 1230 individuals were jointly classified as high risk by both models.

The reclassification pattern between SCORE2 and REGICOR revealed substantial discordance in the identification of workers at high cardiovascular risk (Figure 3).

The marked asymmetry observed in the reclassification pattern suggests that SCORE2 identifies a substantially larger subgroup of workers who may be considered candidates for intensified cardiovascular prevention compared with REGICOR.

### 3.5. Agreement Between SCORE2 and Other Cardiovascular Risk Models

Agreement analyses confirmed that the concordance between SCORE2 and REGICOR was very low. The Cohen’s kappa coefficient for high-risk classification between SCORE2 and REGICOR was 0.094, indicating slight agreement.

By contrast, SCORE2 showed higher agreement with several other models. The strongest agreement was observed between SCORE2 and the Framingham hard coronary events score (kappa = 0.567), followed by Globorisk (kappa = 0.534). SCORE2 demonstrated moderate agreement with ERICE (kappa = 0.439) and fair agreement with DORICA (kappa = 0.357) and the Framingham categorical score (kappa = 0.304).

REGICOR showed consistently low agreement with the remaining cardiovascular risk models. Agreement remained slight with ERICE (kappa = 0.063), Framingham hard coronary events (kappa = 0.080), Globorisk (kappa = 0.081), and the Framingham categorical score (kappa = 0.032). The highest concordance involving REGICOR was observed with DORICA, although agreement remained limited (kappa = 0.186).

These findings indicate substantial heterogeneity in high-risk classification across cardiovascular risk models, with REGICOR showing particularly limited concordance with the remaining scales.

**Figure 4:**
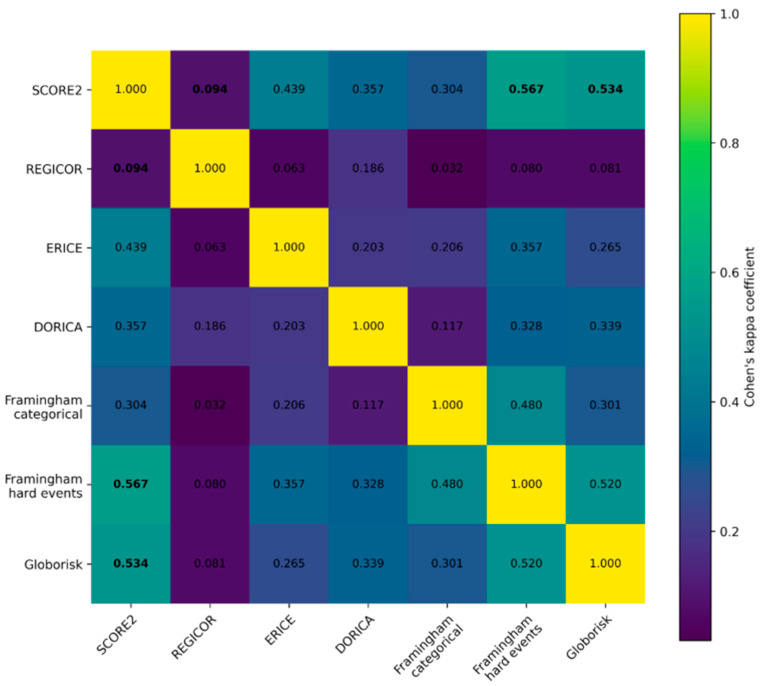
Heatmap of Cohen’s kappa coefficients between cardiovascular risk prediction models. Heatmap showing pairwise agreement between cardiovascular risk prediction models for high-risk classification using Cohen’s kappa coefficients. Darker shades indicate stronger agreement. SCORE2 showed moderate agreement with Globorisk and Framingham hard coronary events scores, whereas agreement between SCORE2 and REGICOR was slight (kappa = 0.094).

Agreement analyses revealed substantial variability in concordance between cardiovascular risk prediction models, with particularly low agreement between SCORE2 and REGICOR (Figure 4).

REGICOR consistently showed limited agreement with the remaining cardiovascular risk models, whereas SCORE2 demonstrated the highest concordance with Framingham hard coronary events and Globorisk.

Pairwise agreement analyses confirmed substantial heterogeneity in concordance across cardiovascular risk prediction models, as detailed in Table 5.

Agreement between SCORE2 and REGICOR was slight (kappa = 0.094), indicating minimal concordance in high-risk classification between both models. In contrast, SCORE2 demonstrated moderate agreement with several contemporary prediction tools, particularly the Framingham hard coronary events and Globorisk equations.

### 3.6. Correlation Between Continuous Cardiovascular Risk Estimates

Correlation analyses showed a different pattern from categorical agreement analyses. SCORE2 showed strong positive correlations with several continuous risk estimates, including the Framingham categorical score (r = 0.768), Framingham hard coronary events score (r = 0.758), Globorisk (r = 0.739), DORICA (r = 0.738), and ERICE (r = 0.715).

In contrast, the correlation between SCORE2 and REGICOR was low (r = 0.251). REGICOR also showed consistently weaker correlations with the remaining cardiovascular risk models, including ERICE (r = 0.212), Globorisk (r = 0.260), Framingham hard coronary events (r = 0.305), Framingham categorical score (r = 0.320), and DORICA (r = 0.340).

The highest correlations were observed among Framingham-derived or conceptually related models. The correlation between the Framingham categorical score and the Framingham hard coronary events score was particularly strong (r = 0.984). DORICA also showed very strong correlations with both the Framingham categorical score (r = 0.956) and the Framingham hard coronary events score (r = 0.936).

These findings indicate that strong correlations between continuous cardiovascular risk estimates do not necessarily translate into clinically meaningful agreement in high-risk classification. This was particularly evident for SCORE2, which showed strong correlations with several scales but only moderate or limited agreement in high-risk classification.

**Figure 5:**
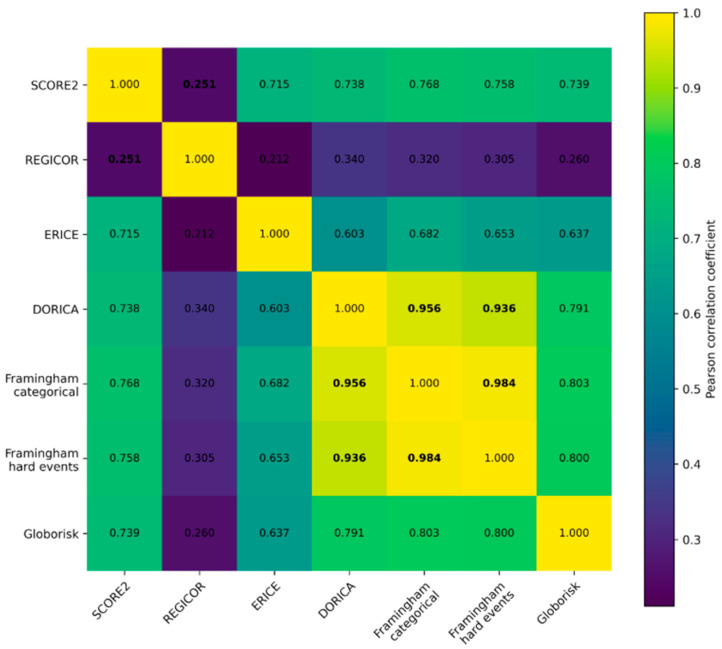
Heatmap of Pearson correlation coefficients between continuous cardiovascular risk estimates. Heatmap showing pairwise Pearson correlation coefficients between continuous cardiovascular risk estimates generated by different cardiovascular risk prediction models. Darker shades indicate stronger positive correlations. SCORE2 showed strong correlations with several contemporary models, whereas correlations involving REGICOR were consistently weaker.

Correlation analyses demonstrated substantial variability in the strength of association between continuous cardiovascular risk estimates generated by the different prediction models (Figure 5).

Pairwise Pearson correlation coefficients between continuous cardiovascular risk estimates are summarized in Table 6.

### 3.7. Integrated Interpretation of the Results

Taken together, the results show that the choice of cardiovascular risk model substantially modified the proportion of workers classified as having high cardiovascular risk. SCORE2 identified a considerably larger high-risk group than REGICOR, despite both models being applied to the same individuals.

The most clinically relevant finding was the limited overlap between SCORE2 and REGICOR in the identification of high-risk individuals. More than nine out of ten workers classified as high risk by SCORE2 were not classified as high risk by REGICOR. This discordance was not explained by a general absence of association between risk models, since SCORE2 showed moderate-to-strong correlations and higher agreement with several other scales.

Therefore, the results support the hypothesis that SCORE2 and REGICOR are not interchangeable tools for cardiovascular risk stratification in Spanish workers. The observed discordance suggests that continued use of REGICOR and SCORE2 may result in substantial differences in cardiovascular risk classification and in the identification of workers categorized as high cardiovascular risk.

However, because the present study was cross-sectional and did not include longitudinal follow-up for cardiovascular outcomes, these findings should be interpreted as evidence of discordance in cardiovascular risk classification rather than proof of superior prognostic performance of any specific model.

## 4. Discussion

### 4.1. Principal Findings

The present study demonstrates substantial discordance between contemporary and traditional cardiovascular risk prediction models when applied to a large cohort of Spanish workers without established cardiovascular disease. The most relevant finding was the marked discrepancy observed between SCORE2 and REGICOR in the identification of individuals classified as high cardiovascular risk. Although both tools are currently used in cardiovascular prevention strategies within Spain, their concordance proved remarkably limited, indicating that they are far from interchangeable in routine clinical practice.

This discordance is likely explained by important methodological differences between the two models, including their underlying cardiovascular endpoints, calibration procedures, and the risk thresholds used to define high-risk categories. SCORE2 estimates both fatal and non-fatal cardiovascular events using contemporary European cohorts, whereas REGICOR is a recalibrated Framingham-based equation focused primarily on coronary outcomes in the Spanish population.

Several clinically relevant observations emerged from the present analysis. First, SCORE2 identified a considerably larger proportion of workers as high risk than REGICOR. Second, agreement in clinically relevant risk categorization differed substantially across models. Third, REGICOR showed the weakest concordance with the remaining cardiovascular risk equations, whereas SCORE2 demonstrated closer alignment with Framingham-derived models and Globorisk. Finally, the observed discordance persisted despite the large sample size and the homogeneous occupational nature of the cohort, reinforcing the robustness and clinical relevance of these findings.

Although SCORE2 identified a substantially larger proportion of workers as high cardiovascular risk, this finding should be interpreted with caution. A broader high-risk classification may improve the identification of individuals who could benefit from preventive interventions, but it may also increase the possibility of overclassification and potentially lead to overtreatment if cardiovascular risk estimates are considered in isolation. Therefore, contemporary preventive strategies increasingly recommend that cardiovascular risk scores be integrated with clinical judgment, comorbidities, risk modifiers, residual cardiovascular risk, and patient preferences when making treatment decisions.

Collectively, these results suggest that the transition from older coronary-focused prediction tools toward newer cardiovascular risk estimation strategies may substantially modify cardiovascular prevention eligibility in Spanish working populations. The findings additionally highlight the growing importance of calibration methodology, endpoint selection, and population-specific risk modeling in contemporary cardiovascular prevention strategies [33,34,35].

### 4.2. SCORE2 and the Paradigm Shift in Cardiovascular Risk Estimation

The publication of SCORE2 represented a major conceptual shift in European cardiovascular prevention because it replaced the original SCORE model and expanded prediction from fatal cardiovascular disease alone toward combined fatal and non-fatal cardiovascular events [5]. This modification substantially increased estimated cardiovascular risk across many European populations and altered thresholds for preventive intervention.

Unlike previous risk equations, SCORE2 was specifically recalibrated using contemporary European cohorts and competing-risk-adjusted statistical methodology [5,33]. Importantly, the model incorporates age-specific risk thresholds and regional recalibration according to cardiovascular mortality patterns across Europe. Spain was classified within the low-risk European region, although important heterogeneity likely persists within different Spanish subpopulations and occupational environments.

Our findings strongly suggest that SCORE2 identifies substantially more workers as high-risk than REGICOR. This observation is consistent with previous reports demonstrating that SCORE2 tends to generate higher cardiovascular risk estimates compared with older equations developed under different epidemiological contexts [34,35]. Several factors may explain this phenomenon.

First, SCORE2 predicts total cardiovascular disease rather than coronary events alone. Second, the contemporary incidence of non-fatal cardiovascular disease remains considerably higher than fatal cardiovascular mortality in modern populations due to improvements in acute cardiovascular care and secondary prevention [36]. Third, the inclusion of competing-risk modeling improves risk estimation in aging populations and modifies absolute risk distributions [33].

These methodological differences likely explain why SCORE2 demonstrated greater concordance with Framingham-derived equations and Globorisk than with REGICOR. In contrast, REGICOR remains fundamentally derived from coronary-focused Framingham recalibration approaches developed under older epidemiological conditions [37].

From a clinical perspective, this divergence may have major implications because individuals classified as low-risk according to REGICOR may simultaneously fulfill high-risk criteria under SCORE2-based European prevention recommendations.

An additional consideration relates to the potential impact of SCORE2 implementation on healthcare-resource utilization. Because SCORE2 identified a substantially larger proportion of workers as high cardiovascular risk, wider adoption of this model in occupational health settings could increase the number of individuals referred for preventive evaluation, follow-up, lifestyle interventions, and pharmacological treatment. Although such an approach may improve early cardiovascular risk management, it could also increase healthcare costs and resource demands. Future studies should evaluate the cost-effectiveness and long-term clinical impact of SCORE2-guided prevention strategies in working populations.

### 4.3. Limited Concordance Between REGICOR and Contemporary Cardiovascular Risk Models

One of the most striking findings of the present study was the consistently poor concordance observed between REGICOR and virtually all other evaluated cardiovascular risk scales. Agreement between REGICOR and SCORE2 was particularly limited despite the fact that both tools are frequently considered alternative approaches for cardiovascular risk assessment in Spain.

This observation deserves careful consideration because REGICOR has historically played an important role in cardiovascular prevention within Spanish primary care settings [37,38]. REGICOR was originally developed through recalibration of the Framingham coronary risk equation using Girona population data in order to adapt cardiovascular risk estimation to the comparatively lower coronary event incidence observed in Mediterranean populations [37].

Although this recalibration improved local applicability at the time of development, several limitations may currently affect its performance in contemporary populations. First, REGICOR was derived primarily from coronary endpoints and does not incorporate broader cardiovascular outcomes. Second, the epidemiological context from which REGICOR emerged differs substantially from current cardiovascular prevention scenarios characterized by aging populations, widespread statin use, improved hypertension management, and declining cardiovascular mortality [36]. Third, occupational cohorts may exhibit cardiovascular risk distributions that differ from those of the original REGICOR derivation population.

The present results indicate that REGICOR and contemporary European prediction strategies classify a substantial proportion of individuals into different cardiovascular risk categories. Similar concerns have recently been raised regarding potential underestimation of cardiovascular risk by older recalibrated equations in certain modern populations [39,40].

Importantly, the poor kappa agreement observed in our study contrasts with the moderate-to-strong correlations identified between several continuous risk estimates. This discrepancy reinforces the concept that statistical correlation does not necessarily imply clinical interchangeability. Two models may correlate reasonably well across a population while simultaneously classifying individual subjects into substantially different therapeutic categories [41].

This issue is especially relevant in preventive cardiology because treatment decisions are generally based on predefined risk thresholds rather than continuous mathematical relationships.

### 4.4. Correlation Does Not Imply Clinical Agreement

One of the most methodologically important observations of the present study was the marked divergence between correlation coefficients and clinical agreement in high-risk classification.

Several cardiovascular risk equations demonstrated strong correlations in continuous risk estimates, particularly among Framingham-derived models and SCORE2. However, these statistical associations translated poorly into concordance when clinically meaningful high-risk categories were analyzed.

This finding has important methodological implications because cardiovascular risk equations are frequently compared using correlation analyses alone. Although correlation coefficients provide useful information regarding the linear relationship between continuous variables, they do not evaluate agreement or interchangeability between diagnostic or predictive tools [41,42]. Consequently, two models may exhibit strong correlation while producing clinically discordant classifications near therapeutic decision thresholds.

Our findings clearly illustrate this phenomenon. SCORE2 showed relatively strong correlations with multiple scales but only moderate or fair agreement in high-risk categorization. Conversely, REGICOR displayed consistently weaker correlations and minimal concordance with the remaining models.

These observations reinforce previous methodological recommendations emphasizing that agreement analyses, calibration assessment, and reclassification metrics should complement correlation-based comparisons when evaluating cardiovascular prediction tools [43,44].

From a practical perspective, reliance exclusively on correlation analyses may substantially overestimate the apparent equivalence between cardiovascular risk equations and potentially obscure clinically meaningful differences in preventive treatment allocation.

### 4.5. Clinical Implications for Cardiovascular Prevention in Spain

The present findings may have important implications for cardiovascular prevention strategies in Spain, particularly within occupational and primary care settings.

Current European prevention guidelines strongly support the use of SCORE2 for cardiovascular risk estimation in individuals without established cardiovascular disease [1,2]. Nevertheless, REGICOR continues to be widely used in multiple Spanish clinical environments because of its historical adaptation to Mediterranean populations and its longstanding incorporation into local prevention protocols.

Our results suggest that continued reliance on REGICOR may lead to different identification of workers considered eligible for intensified preventive strategies according to SCORE2-based recommendations. This discrepancy could potentially affect initiation of lipid-lowering therapy, blood pressure management intensity, lifestyle intervention prioritization, and long-term cardiovascular monitoring.

The issue may be especially relevant in occupational health programs because working populations frequently undergo periodic cardiovascular screening aimed at early prevention. Underestimation of cardiovascular risk in these settings could delay preventive interventions during potentially modifiable stages of disease progression.

At the same time, broader identification of high-risk individuals through SCORE2-based approaches raises important considerations regarding healthcare resource utilization, preventive pharmacotherapy exposure, and possible overmedicalization. Therefore, the optimal balance between sensitivity and specificity in cardiovascular prevention remains an area of ongoing debate [45].

Rather than supporting indiscriminate replacement of one model by another, the present findings highlight the need for careful validation of cardiovascular prediction tools within contemporary Spanish populations and reinforce the importance of understanding the methodological foundations underlying each equation.

### 4.6. Strengths and Limitations

The present study has several important strengths. First, the very large sample size provided substantial statistical power and allowed robust comparison between multiple cardiovascular risk equations. Second, the homogeneous occupational cohort reduced variability related to healthcare access and facilitated standardized cardiovascular risk assessment. Third, the simultaneous evaluation of agreement and correlation metrics allowed a more comprehensive methodological interpretation than studies relying exclusively on single statistical approaches.

Nevertheless, several limitations should also be acknowledged. The cross-sectional design precludes evaluation of actual cardiovascular outcomes and therefore does not permit determination of which prediction model provides superior prognostic accuracy. Longitudinal follow-up studies evaluating incident cardiovascular events are required to clarify the clinical implications of the observed discordance.

Additionally, the occupational nature of the cohort may limit extrapolation to populations with substantially different socioeconomic, ethnic, or clinical characteristics. Workers undergoing occupational health evaluations may also present a healthy worker effect that could influence absolute cardiovascular risk distributions [46].

Furthermore, some evaluated cardiovascular risk equations were originally developed using different cardiovascular endpoints, derivation cohorts, and statistical methodologies. Consequently, direct comparison between scales inevitably involves conceptual heterogeneity beyond purely mathematical differences.

An important limitation of the present study is its cross-sectional design, which precludes evaluation of actual cardiovascular outcomes. Consequently, although substantial differences in risk classification were observed between SCORE2 and REGICOR, the present analysis cannot determine whether either model provides superior prediction of future cardiovascular events. Our findings should therefore be interpreted as evidence of discordance in cardiovascular risk categorization rather than proof of prognostic superiority. Prospective longitudinal studies including hard cardiovascular outcomes are required to establish the comparative predictive performance of these risk equations.

Furthermore, the findings should be interpreted in light of the healthy worker effect, a well-recognized epidemiological phenomenon whereby employed individuals generally exhibit lower morbidity and mortality than the general population. Consequently, the cardiovascular risk distribution observed in this cohort may differ from that of elderly individuals, unemployed populations, or patients with a higher burden of chronic disease. Therefore, caution is warranted when extrapolating the present findings beyond occupational settings.

Despite these limitations, the consistency of the observed discordance across multiple complementary analyses strongly supports the robustness and potential clinical relevance of the findings.

### 4.7. Future Perspectives

Future longitudinal investigations should evaluate the prognostic performance of SCORE2, REGICOR, and other cardiovascular risk equations for predicting actual cardiovascular events in contemporary Spanish populations. Such studies are essential to determine whether the greater sensitivity of SCORE2 translates into improved cardiovascular prevention or alternatively increases overclassification without substantial clinical benefit.

Future investigations should also assess whether the degree of discordance between SCORE2 and REGICOR varies according to sex, age categories, and other clinically relevant demographic subgroups.

Further research should also explore whether recalibration of contemporary cardiovascular prediction tools specifically within Spanish occupational cohorts may improve discrimination and calibration performance.

Additionally, emerging approaches incorporating imaging biomarkers, polygenic risk scores, artificial intelligence-based prediction algorithms, and multidimensional cardiometabolic profiling may further refine individualized cardiovascular risk stratification beyond conventional equations alone [47,48,49].

Overall, the present findings support the growing transition toward more dynamic and population-adapted cardiovascular prevention strategies capable of integrating contemporary epidemiology, competing risks, and individualized risk trajectories.

## 5. Conclusions

In this large cohort of Spanish workers, substantial discordance was observed between SCORE2 and REGICOR in the classification of high cardiovascular risk. SCORE2 consistently identified a considerably larger proportion of individuals as high-risk, whereas REGICOR showed limited concordance with contemporary cardiovascular prediction models.

Although several cardiovascular risk equations demonstrated moderate-to-strong correlations in continuous risk estimates, agreement in clinically relevant high-risk categorization remained substantially lower, reinforcing that correlation does not imply clinical interchangeability.

These findings suggest that reliance on REGICOR instead of SCORE2 may result in substantial differences in cardiovascular risk classification. However, prospective studies evaluating actual cardiovascular outcomes are required before conclusions can be drawn regarding the comparative prognostic performance and clinical superiority of either model.

The results additionally highlight the need for ongoing validation and recalibration of cardiovascular prediction tools in modern Spanish populations and support the transition toward more contemporary, multidimensional, and population-adapted cardiovascular prevention approaches.

## Figures and Tables

**Table 1:** Baseline characteristics of the study population.

Variable	Overall (n = 216,310)	Men (n = 131,260)	Women (n = 85,050)	*p* Value
Age, years	48.8 ± 6.2	49.1 ± 6.1	48.3 ± 6.3	<0.001
Current smokers, n (%)	70,301 (32.5)	42,659 (32.4)	27,642 (32.5)	0.408
Body mass index, kg/m^2^	27.0 ± 4.7	27.6 ± 4.2	26.2 ± 5.0	<0.001
Systolic blood pressure, mmHg	127.6 ± 17.2	131.4 ± 16.8	121.8 ± 16.9	<0.001
Diastolic blood pressure, mmHg	78.8 ± 11.1	81.2 ± 10.8	75.3 ± 10.9	<0.001
Total cholesterol, mg/dL	204.0 ± 36.7	204.8 ± 36.9	202.7 ± 36.3	<0.001
HDL cholesterol, mg/dL	51.3 ± 9.3	48.6 ± 8.1	55.5 ± 8.7	<0.001
LDL cholesterol, mg/dL	129.0 ± 34.9	129.8 ± 35.2	127.7 ± 34.4	0.487
Triglycerides, mg/dL	101 (74–143)	115 (83–162)	85 (65–113)	<0.001
Fasting glucose, mg/dL	94.8 ± 22.1	97.6 ± 23.4	90.6 ± 19.5	<0.001

Data are presented as mean ± standard deviation, median (interquartile range), or number (%), as appropriate. HDL: high-density lipoprotein; LDL: low-density lipoprotein. Continuous variables were compared using Student’s t test or Mann–Whitney U test, as appropriate, whereas categorical variables were compared using the χ^2^ test.

**Table 2:** Distribution of continuous cardiovascular risk estimates according to each prediction model.

Cardiovascular Risk Model	Mean ± SD (%)	Median (IQR) (%)	Minimum (%)	Maximum (%)
SCORE2	1.25 ± 1.96	1.0 (0.0–2.0)	0.0	25.0
REGICOR	3.29 ± 2.30	3.0 (2.0–4.0)	0.5	30.0
ERICE	5.22 ± 5.12	3.0 (2.0–7.0)	0.0	43.0
DORICA	6.28 ± 4.92	5.0 (3.0–8.0)	0.5	69.0
Framingham categorical score	7.62 ± 6.15	7.0 (3.0–10.0)	1.0	63.0
Framingham hard coronary events score	5.29 ± 5.21	4.0 (2.0–7.0)	0.0	65.0
Globorisk	4.21 ± 3.26	3.0 (2.0–6.0)	0.5	20.0

SD: standard deviation; IQR: interquartile range. Continuous cardiovascular risk estimates are expressed as predicted 10-year cardiovascular risk percentages according to each model.

**Table 3:** Number and percentage of individuals classified as high cardiovascular risk according to each prediction model.

Cardiovascular Risk Model	High-Risk Individuals, n	High-Risk Individuals, %
SCORE2	15,617	7.22
REGICOR	4409	2.04
ERICE	12,632	5.84
DORICA	5405	2.50
Framingham categorical score	63,309	29.27
Framingham hard coronary events score	25,001	11.56
Globorisk	16,050	7.42

High cardiovascular risk was defined according to the thresholds recommended for each specific prediction model.

**Table 4:** Cross-classification of high cardiovascular risk according to SCORE2 and REGICOR.

	REGICOR Non-High Risk	REGICOR High Risk	Total
SCORE2 non-high risk	197,514	3179	200,693
SCORE2 high risk	14,387	1230	15,617
Total	211,901	4409	216,310

High cardiovascular risk categories were defined according to the thresholds recommended for each cardiovascular risk model. Agreement between SCORE2 and REGICOR for high-risk classification showed a Cohen’s kappa coefficient of 0.094, indicating slight agreement.

**Table 5:** Pairwise agreement between cardiovascular risk prediction models for high-risk classification. Values represent Cohen’s kappa coefficients for agreement in high cardiovascular risk classification between cardiovascular risk prediction models.

	SCORE2	REGICOR	ERICE	DORICA	Framingham Categorical	Framingham Hard Events	Globorisk
SCORE2	1.000	0.094	0.439	0.357	0.304	0.567	0.534
REGICOR	0.094	1.000	0.063	0.186	0.032	0.080	0.081
ERICE	0.439	0.063	1.000	0.203	0.206	0.357	0.265
DORICA	0.357	0.186	0.203	1.000	0.117	0.328	0.339
Framingham categorical	0.304	0.032	0.206	0.117	1.000	0.480	0.301
Framingham hard events	0.567	0.080	0.357	0.328	0.480	1.000	0.520
Globorisk	0.534	0.081	0.265	0.339	0.301	0.520	1.000

Values represent Cohen’s kappa coefficients for agreement in high cardiovascular risk classification between cardiovascular risk prediction models. Kappa coefficients were interpreted according to the Landis and Koch classification: <0.20, slight agreement; 0.21–0.40, fair agreement; 0.41–0.60, moderate agreement; 0.61–0.80, substantial agreement; >0.80, almost perfect agreement.

**Table 6:** Pairwise Pearson correlation coefficients between continuous cardiovascular risk estimates.

	SCORE2	REGICOR	ERICE	DORICA	Framingham Categorical	Framingham Hard Events	Globorisk
SCORE2	1.000	0.251	0.715	0.738	0.768	0.758	0.739
REGICOR	0.251	1.000	0.212	0.340	0.320	0.305	0.260
ERICE	0.715	0.212	1.000	0.603	0.682	0.653	0.637
DORICA	0.738	0.340	0.603	1.000	0.956	0.936	0.791
Framingham categorical	0.768	0.320	0.682	0.956	1.000	0.984	0.803
Framingham hard events	0.758	0.305	0.653	0.936	0.984	1.000	0.800
Globorisk	0.739	0.260	0.637	0.791	0.803	0.800	1.000

Values represent Pearson correlation coefficients calculated using continuous cardiovascular risk estimates generated by each prediction model. All correlations were statistically significant (*p* < 0.001).

## Data Availability

Strict measures were implemented to ensure participant confidentiality and data security throughout the entire research process. Prior to analysis, all records were irreversibly anonymised to prevent the identification of individual participants. Data management procedures were performed in accordance with the principles established in the Spanish Organic Law 3/2018 on Personal Data Protection and Guarantee of Digital Rights, as well as the European Union General Data Protection Regulation (GDPR; Regulation EU 2016/679). The datasets generated and analyzed during the current study are maintained in a secure institutional repository at ADEMA University School. Access to fully anonymised data may be considered upon reasonable request to the corresponding author, subject to compliance with applicable ethical requirements and current data protection legislation.
